# Secondary primary tumor mimicking osteoradionecrosis

**DOI:** 10.4322/acr.2021.389

**Published:** 2022-07-22

**Authors:** Giovanna Lopes Carvalho, Daniele Heguedusch, Antônio Cássio Assis Pellizzon, Renan Bezerra Lira, Fábio Abreu Alves, Graziella Chagas Jaguar

**Affiliations:** 1 A.C. Camargo Cancer Center, Departamento de Estomatologia, São Paulo, SP, Brasil; 2 A. C. Camargo Cancer Center, Departamento de Radioterapia, São Paulo, Brasil; 3 A.C. Camargo Cancer Center, Departamento de Cirurgia de Cabeça e Pescoço e Otorrinolaringologia, São Paulo, SP, Brasil.

**Keywords:** Nasopharyngeal carcinoma, second primary neoplasms, squamous cell carcinoma of head and neck, osteoradionecrosis, case reports

## Abstract

Nasopharyngeal carcinoma (NPC) is a malignant tumor rarely found in the head and neck, representing about 1% of all malignancies. The main treatment for NPC is radiation therapy, which is often given in combination with chemotherapy. However, such treatment may lead to long‐term complications, including second primary tumors (SPTs) and osteoradionecrosis (ORN). Both complications have similar radiological characteristics, which can lead to erroneous diagnoses. This paper describes a case of a second primary tumor in a patient after 20 years of radiotherapy in the area where a previous extraction was performed, mimicking an osteoradionecrosis process.

## INTRODUCTION

Nasopharyngeal carcinoma (NPC) is an epithelial carcinoma arising from the nasopharyngeal mucosal lining.^1^ It is a rare malignancy, but approximately 80% of diagnosed patients are from Asia.^1^ Radiotherapy is the main therapeutic modality for NPC; however, such treatment may lead to long‐term complications, including second primary tumors (SPTs) and osteoradionecrosis (ORN).

According to the literature, the incidence of SPTs after radiotherapy treatment in patients with NPC ranges from 0.82 to 5.6%.^2^^,^ 3 ^,^ 4 Sarcoma and squamous cell carcinoma (SCC) are the most common histological subtypes of radio-induced tumors in the head and neck. Some authors report that specifically in patients after radiotherapy for NPC, the most common is SCC.^4^ Due to unusual clinical features, these radio-induced tumors can be misdiagnosed,^5^ especially with ORN, because of similar radiological characteristics.

Here, we described a case of SPTs mimicking ORN after 20 years of radiotherapy for NPC and highlighted the importance of cancer patients maintaining long follow-ups to avoid diagnostic delays.

## CASE REPORT

**Figure 1:**
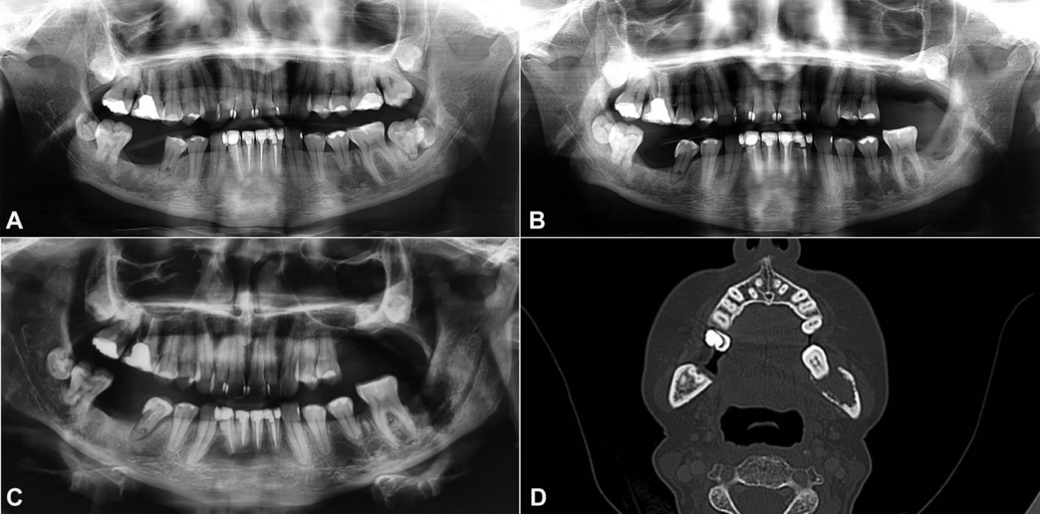
**A** – initial panoramic radiography made for diagnosis of the pain in 26, 27 and 37 in 2017 showing alveolar bone loss in the region of 26 and 27, and element 37 with abnormal shape and presence of radiolucent image around; **B** – a panoramic made for follow the healing of the alveolus, 10 months after tooth extraction of 37, showing an alveolar bone repair; **C** – panoramic radiography made in 2020 after the complained of bleeding, showing an irregular and diffuse radiolucent area in the posterior region of the mandible; **D** – CT of the head, in axial section showing ill-defined hypodense area in alveolar process of the left mandible in the region of 37 tooth, with rupture of the cortical bone and bone sequestration areas.

The patient reported in this article has signed informed consent for publication. A 34-year-old woman attended the Stomatology Department with a complaint of sporadic bleeding in the left mandible over the last 6 months. Her medical history included the diagnosis of nasopharyngeal lymphoepithelioma at age 9 (T2N2M0), being treated with 25 fractions of 2D-radiotherapy in the nasal cavity and bilateral cervical regions totalizing 4500 cGy, a booster dose of 5 fractions in nasopharynx and neck (1000 cGy) and brachytherapy in 2 fractions (1000 cGy) in nasopharynx. Furthermore, she completed 6 cycles of chemotherapy with cisplatin (3 cycles before radiotherapy and 3 cycles after the end of brachytherapy). The patient had no relevant family history of cancer and had not undergone germline genetic testing. Soon after the end of the oncological treatment, the patient progresses with severe trismus and dry mouth, which caused very compromised oral hygiene, radiation-related caries, and other dental issues in the following years, prompting the need for various dental treatments. In 2017, she had multiple extractions in the maxilla and mandibula at different times because of tooth mobility, caries, and pain refractory to more conservative treatments. No histological analysis of the teeth was performed, as there were no significant alterations. Due to the risk of ORN development, the patient maintained semiannual appointments to monitor the complete healing of the alveolus. (Figure 1A-B).

The patient returned for an in-person visit almost 1 year after the last appointment. The intra-oral examination was challenging as a result of severe trismus However, it was possible to observe that the currently reported bleeding was originated from the mandibular left permanent second molar alveolus. It was not possible to observe any exposed bone. Panoramic radiography was performed and revealed diffuse and undefined radiolucency in the posterior region of the mandible (Figure 1C). Computed tomography (CT) scan of the area showed a hypodense structure in the alveolar process of the left mandible in the region of the mandibular left permanent second molar (Figure 1D).

According to imaging findings, our first hypothesis was ORN in the region where extraction was performed 3 years ago. However, as there was no exposed bone and infectious signs, the diagnosis of a second primary tumor was also likely. An incisional biopsy was performed, which revealed a squamous cell carcinoma (SCC).

**Figure 2:**
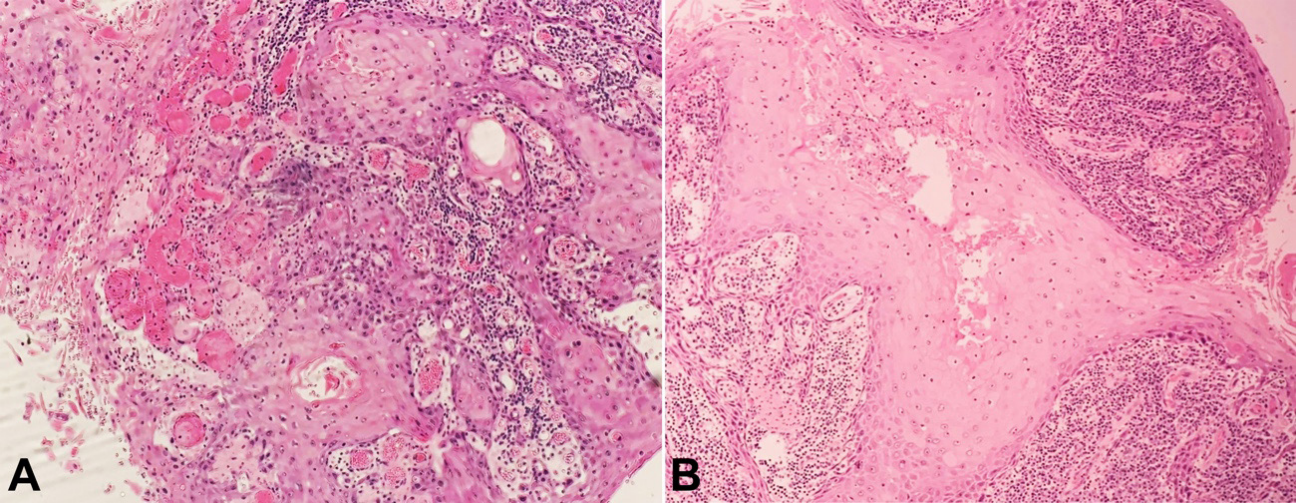
**A** and **B** – Histopathology showing a well-differentiated SCC (H&E, 40X).

The patient was referred to the Head and Neck Surgery Department and underwent left segmental mandibulectomy, selective neck dissection, and the mandible and oral cavity reconstruction with osteocutaneous flap of the left fibula. The histopathologic report showed a well-differentiated SCC of 1.8 x 1.8 cm, with an infiltration depth of 0.8 cm and infiltration in the lingual nerve (Figure 2A and 2B).

**Figure 3:**
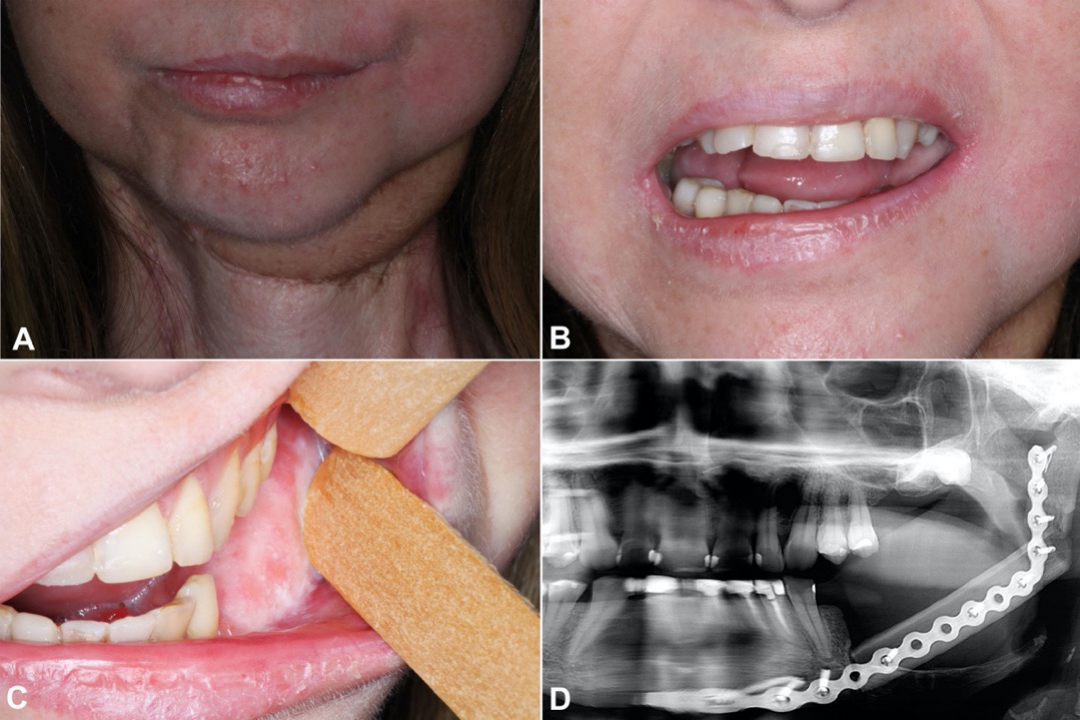
**A-C** Photography of follow-up 5 months after surgery; **D** – panoramic radiography made after the segmental mandibulectomy showing the reconstruction of the mandible.

The section margins and lymph nodes were free of tumor (pT4aN0). The patient was submitted to new radiotherapy (IMRT technique) with 5 fractions, totalizing 2500cGy in the tumor area, and maintained monthly follow-up, free of new lesions. (Figure 3A-D).

**Figure 4:**
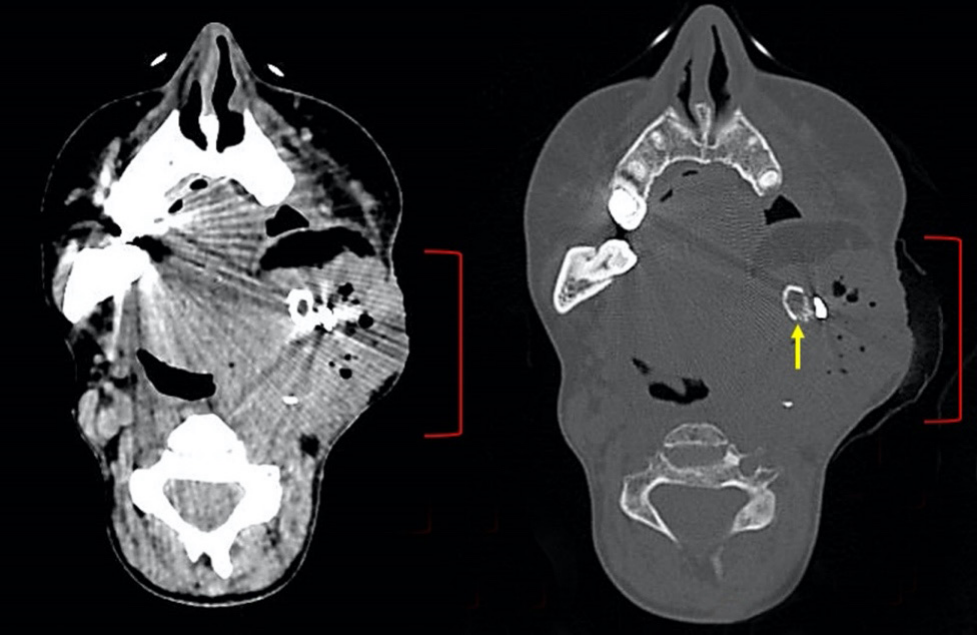
CT of the head, in axial section showing a poorly delimited lesion located in the left masticatory space/flap margins, with gaseous foci intermingling, with apparent extension to the skin of the malar region, pterygoid plate, nasopharynx, soft palate, carotid space, deep portion of the left parotid (red brackets), a diffuse change in the bone texture of the distal left mandibular stump/coronoid process, with bone erosions with cortical rupture (yellow arrow), and lymph node enlargement in the cervical and paratracheal region, favoring the diagnosis of locoregional recurrence and tumor site infection.

Nine months after the end of radiotherapy, the patient presented with edema in the left cervical and facial region, without fever or pain, with purulent secretion coming out of an extra-oral fistula in the left submandibular region (under the surgical scar). The first hypothesis was an infection of the synthesis material or osteomyelitis. Ciprofloxacin was prescribed for 7 days and a CT scan was requested. The CT scan showed an expansive lesion in the left masticatory space and lower cervical lymph nodes that were also suspected of secondary neoplastic involvement (Figure 4).

The MRI later showed an expansive and ulcerated lesion measuring 90 x 61 x 47 mm on the surgical margins with a recurrent neoplastic appearance, with invasion of the larynx, infratemporal fossa, carotid vascular, and intracranial perineural dissemination, in addition to metastatic lymph nodes. A CT-guided biopsy was performed in the right cervical region with the result of moderately differentiated SCC, confirming the hypothesis of an aggressive recurrence with superimposed infection. One week after the consultation, she had a peak fever (37.9ºC) and was hospitalized to start a new antibiotic regimen (ceftriaxone and clarithromycin) and new oncological treatment regimen. Due to the tumor’s size and location and also the relationship with noble structures, the head and neck surgery team considered the case beyond the possibility of surgical resection. The Oncology team was scheduled to start first-line chemotherapy (carboplatin and taxol) after the resolution of the infection. After starting the antibiotic therapy, the patient no longer had fever and maintained stable vital signs, despite having a very high CRP (117 mg/L). During hospitalization, the patient was diagnosed with thrombosis in the internal jugular artery and full anticoagulation was started. Two days after admission, the patient had a cardiorespiratory arrest during sleep, and despite undergoing full resuscitation maneuvers, the patient did not survive. Although death was completely unexpected at that moment, the family did not authorize a full autopsy procedure.

## DISCUSSION

NPC survivors undergoing radiotherapy may experience significant late side effects even many years after the treatment. Since most patients with NPC are diagnosed at a young age and have a long-term survival, SPT attributable to RT has become a major concern 3. The incidence of SPTs in NPC survivors is less than 5%, but some authors have noted that this incidence increased.^3^ According to the literature, SCC is the most common SPT in NPC survivors,^4^ with an incidence ranging from 0.2 to 0.29%.^3^

The mechanism behind SPT is not fully understood. However, two theories have been proposed: “condemned mucosa syndrome”, which claims that cancer cells can migrate in a generalized way to other tissues or organs of the aerodigestive tract, and “field cancerization”, which claims that the mucosa accumulates genetic alterations after prolonged and repeated exposure to a carcinogenic agent, which leads to the induction of multiple and independent malignant lesions^6^. However, both theories are not fully accepted by NPC survivors. According to Kong et al. 2006 in a retrospective study of 326 cases of undifferentiated NPC, 17 patients developed an SPT, and none of them were an undifferentiated carcinoma (same as the nasopharynx), excluding the possibility of condemned mucosa syndrome. Regarding the field of cancerization theory: the only known risk factor for NPC is infection by the Epstein-Barr virus, which is unrelated to oral carcinoma^6^. Therefore, ionizing radiation is more accepted as the main cause. However, other factors associated with the development of SPT in surviving NPC are currently being studied, such as overexpression of epidermal growth factor receptors and the vascular endothelial growth factor^6^.

For a tumor to be considered radio-induced, it must follow the following criteria: occur in a region with a history of radiotherapy, with evidence that there was no tumor in the region before treatment; having a latency period between radiotherapy and the development of the second tumor (authors suggest between 3-5 years at least); be histologically different from the first primary tumor; the patient does not have any syndrome that predisposes to cancer.^7^ Our patient met all criteria, which corroborate the diagnosis of radio-induced tumor.

Due to the rarity and unusual clinical and radiographic characteristics, SPT can be confused with other pathologies, including ORN. ORN is another late side effect of radiotherapy that can occur years after the treatment, especially after some traumatic factor such as tooth extraction.^8^ In this case, the first hypothesis was ORN as it was in the same region that underwent tooth extraction 3 years ago, in a patient who was submitted to radiotherapy treatment in the region. However, what drew attention was the absence of exposed bone and infectious signs, which was decided by biopsy as a way to exclude the possibility of carcinoma, which ended up being confirmed.

NPC survivors with SCC have a worse prognosis than patients with sporadic SCC, with a 5-year overall survival rate of 47% versus 62% for sporadic SCC.^9^ When the TNM classification of NPC survivors with SCC was III/IV, the 5-year survival rate was 13%.^3^ Complete surgical resection is the first-line treatment for SPT in NPC survivors and provides a higher survival rate.^3^ The decision of postoperative re-irradiation is still questionable. However, it is currently recommended for selected high-risk patients who can tolerate treatment toxicity, aiming to increase locoregional control and disease-free survival.^10^ Our patient was considered high-risk and was subjected to re-irradiation. The patient had a locoregional recurrence 9 months after the end of radiotherapy and died 14 months after the initial diagnosis.

In conclusion, we emphasize the aspects of a secondary SCC after 20 years of radiotherapy for NPC mimicking an ORN case, as it occurred at the same site of previous tooth extraction. It is important to add SPT in jawbone lesions as a differential diagnosis when the patient has a previous history of radiotherapy in the region.
